# Transcriptome analysis reveals plant response to colchicine treatment during on chromosome doubling

**DOI:** 10.1038/s41598-017-08391-2

**Published:** 2017-08-17

**Authors:** Kai Zhou, Paige Fleet, Eviatar Nevo, Xinquan Zhang, Genlou Sun

**Affiliations:** 1 Department of Grassland Science, Animal Science and Technology College, Sichuan Agricultural University, Chengdu, 611130 China; 2 Department of Biology, Saint Mary’s University, Halifax, NS B3H3C3 Canada; 3 Institute of Evolution, University of Haifa, Mount Carmel, Haifa 3498838 Israel

## Abstract

Colchicine was commonly used to artificially double chromosomes while the transcriptome changes in colchicine treated plants has rarely been characterized. To understand the molecular mechanism of colchicine on chromosome doubling, we characterized transcriptome data of diploid orchardgrass root after colchicine treatment. Our results showed that 3381 of differentially expressed genes (DEGs) were mainly affected by water stress, 1258 DEGs that were expressed significantly in sample DacR5tr but not in DacR5ck were considered to be mainly affected by colchicine and combination of water and colchicine. These DEGs mainly regulated by colchicine were enriched to gene ontology (GO) accessions of cation binding, catalytic activity, membrane and transporter activity, and enriched to Kyoto Encyclopedia of Genes and Genome (KEGG) pathways of phenylpropanoid biosynthesis, phenylalanine metabolism, plant hormone signal transduction and starch and sucrose metabolism. Genes related to microtubule, spindle, chromosomal kinetochore, vesicle, cellulose and processes of cytoplasm movement, chromatid segregation, membrane and cell wall development were inhibited by colchicine. Our results revealed that colchicine restrained the microtubules and inhibited gene expression of cytokinesis, which might slow down the cell activity, delay the cell into anaerobic respiration, resulting in apoptosis at late stage, and relieving of waterlogging.

## Introduction

Genome or chromosome doubling is a process of doubling complete sets of chromosomes, as the result, a polyploid is usually generated. As reported, polyploidy occurred in about 30% to 70% of flowering plants in nature^1, 2^. Genome duplication events of many different plants supposedly occurred around the Cretaceous-Tertiary^3^. Several mechanisms could induce the formation of polyploids. Polyploidy generated from somatic doubling was commonly found in non-meristematic plant tissues^4^. Different polyploid cells or mixoploid chimeras were produced by somatic doubling^5–8^. Another common pattern of polyploid formation was non-reduction during micro- and mega- gametogenesis^9^, which generated 2n gametes with full somatic chromosomes^10–12^.

After polyploidization, polyploidy was considered to increase the phenotypic variability, vigor, tolerance of abiotic stress and adaptability in novel habitats, even the reproduction modes of plants can be changed^3, 13, 14^. Due to these features, artificial chromosome doubling methods were developed, and several antimitotic agents were often used for doubling chromosomes, including colchicine, trifluralin, oryzalin, amiprophos-methyl, carbamate, etc. Colchicine was the most commonly used agent for inducing polyploidy with embryogenic callus, *in vitro* shoots, shoot apices or leaf explants^15, 16^.

As early as the 1930s, the first application of plant chromosome doubling using colchicine was reported^17^. Subsequently, colchicine solution and other succedaneums were widely applied to generate artificial polyploid plants, and the mechanisms it employed were explored^18–20^. It has been revealed that colchicine binds with alpha- and beta-tubulin dimers^21^, which results in chromosome doubling. However, alpha- and beta-tubulin dimers are the basic element for assembly of microtubules. As already known, microtubules play important roles in the cytoskeleton, and are involved in a number of cellular processes, especially mitosis^22^. During mitosis, microtubules will form spindles, extend the length from spindles to chromosomal kinetochores with continuous supply of alpha- and beta-tubulin dimers, and then pull chromosomes toward two polar ends of the cell. Colchicine can associate with tubulin dimers to reduce the amount of microtubules in the synthesis process^21, 23^, which may prevent spindle formation, shorten the length of spindle fibers, even result in non-division of cells. Although it has widely been accepted that colchicine blocks the cell division by inhibiting the synthesis of microtubules, the exact mechanism on chromosome doubling remains unclear. Furthermore, the mechanism of colchicine was mostly studied on human health, rarely on plants^24, 25^.

*Dactylis* L., commonly known as orchardgrass, belongs to *Festucaea* in the Poaceae family, is an important perennial, cool-season and tall-growing grass, and consists of diploids, tetraploids and a hexaploid. Due to being widely used as forage and hay, *Dactylis* has been spread almost over every continent. Orchardgrass is one of the top four perennial forage grass, with about 14 million pounds of seed harvested each year^26^. To date, *Dactylis* species commonly used as forage were tetraploids, such as *D. glomerata* subsp. *glomerate*, subsp. *hispanica* and subsp. *marina* ^27^. Although there are many diploid species in orchardgrass, many of them are threatened by climate warming and habitat degradation. To supply and expand the gene pool for *Dactylis* breeding programs, diploids have been used for breeding through artificial chromosome doubling of diploids^28–30^.

To double the chromosome of plants using colchicine, the common process is to soak plant tissue in colchicine solution^16, 31^. Water, colchicine, and potential synergism of water and colchicine could cause transcriptome changes in treated individuals, which has rarely been characterized. To explore the mechanism of colchicine on chromosome doubling, and reveal the effect of colchicine on the plant as one stress, diploid orchardgrass, *D. glomerata* subsp. *smithii* L., was treated with colchicine. Transcriptome analysis was performed to reveal the change of gene expression in response to the treatment.

## Results

### Transcriptome assembly and gene annotation

Total of 48999576, 50310774 and 51255434 clean reads were obtained from 51481126, 52867138 and 53823216 raw reads in three *Dactylis* samples of DacR0 (0 h, non-treated), DacR5ck (5 h, water treated) and DacR5tr (5 h, colchicine treated). Full-length of transcriptomes was reconstructed using the clean reads via Trinity because of the lack of a reference genome. Total of 251227 transcripts were finally assembled, of which, 172524 unigenes were selected since those were the longest transcripts of each gene. The length of unigenes ranged from 201 to 15543 base pairs (bp), with mean length of 583 bp, median length of 331 bp. The length of N50 and N90 were 843 bp and 249 bp, respectively.

Among the 172524 unigenes, 84391 genes (48.92%) were annotated in at least one database, of which, 63211, 49178, 21567, 45944, 49387, 50502 and 25391 unigenes were significantly matched to NR, NT, KO, SwissProt, PFAM, GO, and KOG, respectively. The 63211 unigenes annotated in the NR frequently matched with genes of *Brachypodium distachyon* (18.6%), *Aegilops tauschii* (15.5%), *Hordeum vulgare* (14.7%), *Triticum urartu* (7.8%), *Oryza sativa* (4.5%) and others (38.8%).

BLAST search against the NR and SwissProt databases found that, out of the 172524 unigenes, 67653 genes were predicted as CDSs with an average length of 603 bp, ranging from 30 bp to 15102 bp. In addition, ESTScan showed that 92127 unigenes that were not hit in BLAST were predicted as CDSs with an average length of 305 bp, ranging from 51 bp to 6609 bp.

After genes were functionally annotated, the genes were described and estimated according to the biological function via GO project. Biological processes, cellular components and molecular functions were applied to screen the genes’ attributes. Among the unigenes, 50502 genes were attached to at least one GO term. In the biological process term, genes were mainly attached to cellular process (52.82%), metabolic process (51.49%) and single-organism process (38.82%). The cell, cell part and organelle, were found as the first three elements in cellular component with a proportion of 28.67%, 28.65% and 19.34%, respectively. In the molecular function term, the first three major elements were binding (52.66%), catalytic activity (42.40%) and transporter activity (5.86%).

Total of 21567 unigenes were annotated in KEGG database, and were classified into 131 secondary pathways of 5 primaries. Scanning in the secondary pathways, translation of genetic information processing term held most of the unigenes (2989 genes, 13.86%), followed by carbohydrate metabolism of metabolism term (2323 genes, 10.77%).

KOG analysis annotated 25391 unigenes into 26 categories, among which general function prediction contained the most abundant genes (3990 unigenes, 15.71%), followed by posttranslational modification, protein turnover, and chaperones (3746 unigenes, 14.75%).

### DEGs analysis in DacR5tr vs DacR0 and DacR5ck vs DacR0

**Figure 1:**
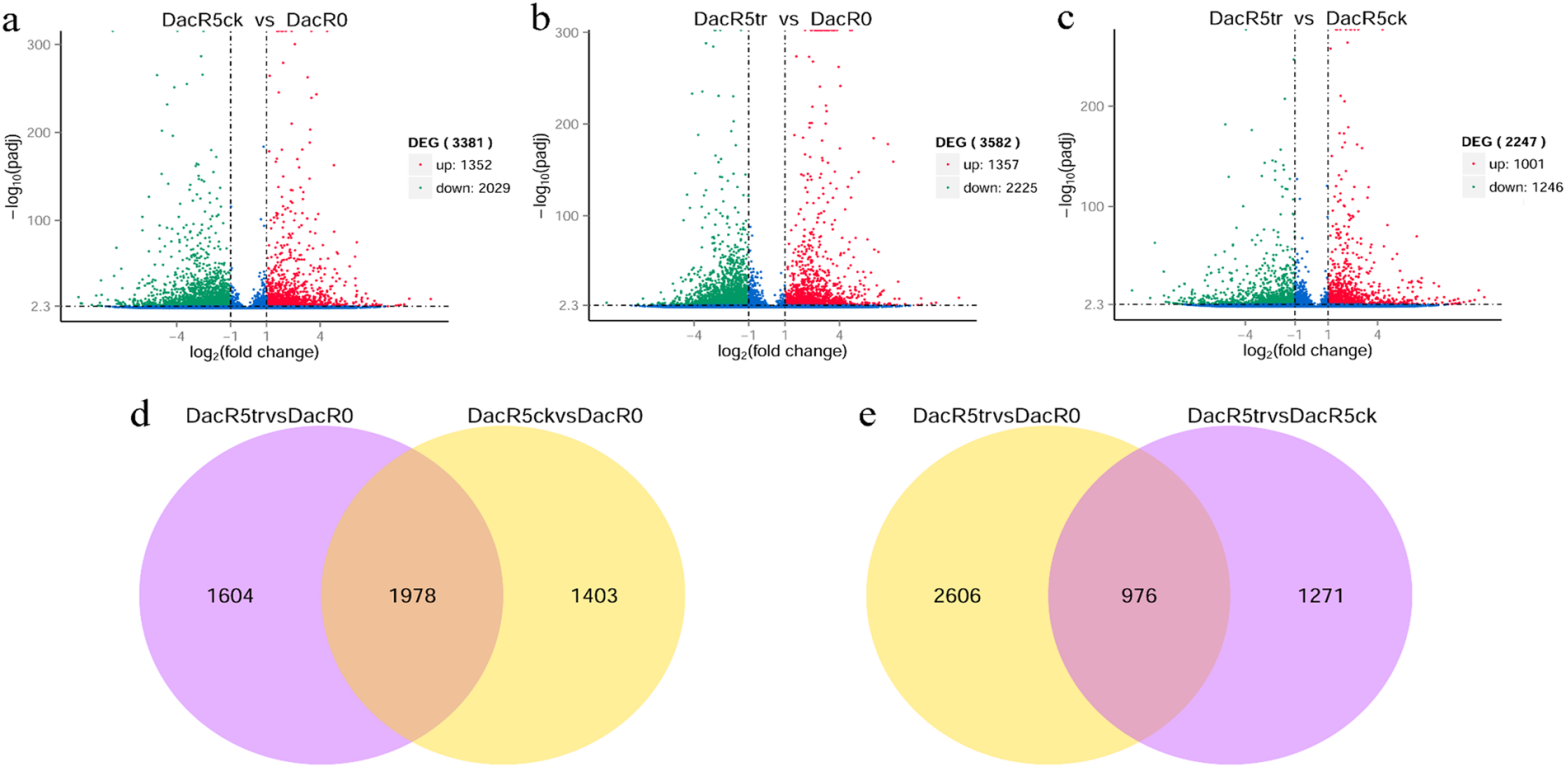
Filtering differentially expressed genes between samples. Volcano plot was used to show the overall distribution of differentially expressed genes (DEGs). In part a, b and c of this figure, gene with |log2 (foldchange)| > 1 and q-value < 0.005 was screened out as significantly DEG. The up-regulated DEGs were represented by red dots, while down-regulated DEGs were represented by green dots. The x-axis means the fold change of DEGs, and the y-axis is the p-value after normalized. The bigger the −log10(padj), the more significant the difference is. Part d and e is the Venn diagram of DEGs between transcriptomes comparisons.

The normalized read count of each unigene was applied to differential expression analysis. After filtering, 3381 and 3582 differentially expressed genes (DEGs) were screened out in comparison of DacR5ck vs DacR0, and DacR5tr vs DacR0, respectively (Fig. 1a,b). Compared with untreated DacR0, 1352 and 1357 DEGs were up-regulated in DacR5ck and DacR5tr, whereas 2029 and 2225 DEGs were down-regulated in DacR5ck and DacR5tr, respectively. Total of 1978 DEGs were common between DacR5ck vs DacR0 and DacR5tr vs DacR0 (Fig. 1c), in which 1361 down-regulated DEGs and 561 up-regulated DEGs were detected in the both comparisons, while 56 DEGs were inconsistent between the two comparisons.

According to the GO enrichment, the first 4 GO categories of DEGs from DacR5ck vs DacR0 and DacR5tr vs DacR0 were metabolic process (1604 genes, 63.78%; 1703 genes, 62.96%), catalytic activity (1408 genes, 55.98%; 1492 genes, 55.16%), single-organism metabolic process (751 genes, 29.86%; 825 genes, 30.5%) and biosynthetic process (700 genes, 27.83%; 767 genes, 28.35%).

KEGG pathway enrichment analysis showed the highest number of DEGs was involved in phenylpropanoid biosynthesis, followed by plant hormone signal transduction and phenylalanine metabolism in DacR5ck vs DacR0. However, the largest part of KEGG pathway in DacR5tr vs DacR0 was ribosome, followed by the phenylpropanoid biosynthesis, starch and sucrose metabolism. In addition, these four pathways were enriched as down-regulation.

The fold change of DEGs showed that two alcohol dihydrogen genes were most upregulated in DacR5ck vs DacR0, followed by the genes related to ATP metabolic process and oxidation-reduction process. The genes with the highest up-regulation in DacR5tr vs DacR0 were related to proteolysis and oxidation-reduction process.

### DEGs analysis in DacR5tr vs DacR5ck

After data normalization and filtering, 2247 DEGs were identified between DacR5tr and DacR5ck. Compared with DacR5ck, 1001 DEGs were up-regulated, and 1246 unigenes were down-regulated in DacR5tr (Fig. 1d).

GO and KEGG enrichment analysis were processed with these DEGs. Total of 2225 DEGs were involved in 2990 GO accessions, among which, DEGs were mainly enriched in biological process and molecular function terms. Among the DEGs, up-regulated DEGs were enriched mainly in cation binding and metal ion binding terms, while down-regulated DEGs were enriched mainly in catalytic activity, membrane and oxidation-reduction process (Table 1). KEGG enrichment analysis showed that DEGs were involved in pathways of plant hormone signal transduction, phenylpropanoid biosynthesis, starch and sucrose metabolism, apoptosis, chemical carcinogenesis, etc. Among the 20 KEGG pathways, 10 were mainly down-regulated and 10 were up-regulated DEGs (Table 2).

**Table 1:** Top several GO enrichments based on DEGs of DacR5htr vs DacR5hck.

GO accession	Term type	Description	Up gene Number	Down gene number
**Up-regulation**				
GO:0043169	molecular function	cation binding	150	120
GO:0046872	molecular function	metal ion binding	148	120
**Down-regulation**				
GO:0055114	biological process	oxidation-reduction process	82	128
GO:0006979	biological process	response to oxidative stress	5	25
GO:0006804	biological process	obsolete peroxidase reaction	4	22
GO:0006555	biological process	methionine metabolic process	1	9
GO:0006857	biological process	oligopeptide transport	0	4
GO:0016020	cellular component	membrane	161	267
GO:0003824	molecular function	catalytic activity	392	497
GO:0016491	molecular function	oxidoreductase activity	85	123
GO:0005215	molecular function	transporter activity	59	102
GO:0046906	molecular function	tetrapyrrole binding	14	43
GO:0020037	molecular function	heme binding	13	43
GO:0016209	molecular function	antioxidant activity	8	25
GO:0016684	molecular function	oxidoreductase activity, acting on peroxide as acceptor	6	22
GO:0004601	molecular function	peroxidase activity	4	22

**Table 2:** Top 20 KEGG pathway enrichments in DacR5htr vs DacR5hck.

KEGG pathway Term	KO ID	Down-regulated DEGs number	Up-regulated DEGs number	Total number
**Down-regulation**				
Phenylpropanoid biosynthesis	ko00940	38	2	40
Phenylalanine metabolism	ko00360	33	2	35
Plant hormone signal transduction	ko04075	17	6	23
Methane metabolism	ko00680	10	5	15
Apoptosis	ko04210	8	2	10
Calcium signaling pathway	ko04020	6	3	9
Flavonoid biosynthesis	ko00941	8	0	8
Stilbenoid, diarylheptanoid and gingerol biosynthesis	ko00945	5	1	6
Flavone and flavonol biosynthesis	ko00944	4	0	4
Degradation of aromatic compounds	ko01220	4	0	4
**Up-regulation**				
Starch and sucrose metabolism	ko00500	13	18	31
Cysteine and methionine metabolism	ko00270	11	10	21
Galactose metabolism	ko00052	6	11	17
alpha-Linolenic acid metabolism	ko00592	3	9	12
Fructose and mannose metabolism	ko00051	3	9	12
Metabolism of xenobiotics by cytochrome P450	ko00980	4	6	10
Drug metabolism - cytochrome P450	ko00982	4	6	10
Chemical carcinogenesis	ko05204	3	6	9
Linoleic acid metabolism	ko00591	1	5	6
Arachidonic acid metabolism	ko00590	1	4	5

### DEGs related to cell division

DEGs were deeply scanned to reveal the expression of genes related to cell division by comparing the transcriptome of DacR5tr vs DacR5ck. The results of GO enrichment showed that more than 170 GO accessions identified involved in cell division processes, which were descripted as organization or processes of microtubule, molecular motor, spindle, kinetochore, cell cycle checkpoint, Golgi, membrane, vesicle, chromosome segregation, cell wall, cell cycle, etc (Part of the GO terms was showed in Table 3).

**Table 3:** Part of the GO terms which were Screened out related to mitosis in cell cycle.

GO_accession	Description	Up-regulated DEGs number	Down-regulated DEGs number
GO:0000910	cytokinesis	0	4
GO:0006928	movement of cell or subcellular component	3	8
GO:0007015	actin filament organization	1	1
GO:0005819	spindle	1	3
GO:0051225	spindle assembly	2	0
GO:0000775	chromosome, centromeric region	6	5
GO:0008608	attachment of spindle microtubules to kinetochore	1	2
GO:0000779	condensed chromosome, centromeric region	1	3
GO:0000776	kinetochore	2	4
GO:0034508	centromere complex assembly	2	2
GO:0051382	kinetochore assembly	2	1
GO:0008017	microtubule binding	1	5
GO:0045298	tubulin complex	1	5
GO:0031109	microtubule polymerization or depolymerization	0	1
GO:0005875	microtubule associated complex	1	2
GO:0005815	microtubule organizing center	2	0
GO:0015630	microtubule cytoskeleton	5	7
GO:0005856	cytoskeleton	10	12
GO:0008092	cytoskeletal protein binding	16	13
GO:0005794	Golgi apparatus	3	6
GO:0000139	Golgi membrane	1	3
GO:0030660	Golgi-associated vesicle membrane	0	2
GO:0031975	envelope	9	19
GO:0016192	vesicle-mediated transport	8	10
GO:0016023	cytoplasmic membrane-bounded vesicle	2	8
GO:0000785	chromatin	5	2
GO:0000280	nuclear division	6	3
GO:0000819	sister chromatid segregation	0	1
GO:0007059	chromosome segregation	3	4
GO:0051301	cell division	2	6
GO:0061640	cytoskeleton-dependent cytokinesis	0	4
GO:0000281	mitotic cytokinesis	0	4
GO:0007049	cell cycle	9	13
GO:0000278	mitotic cell cycle	3	5
GO:0000279	M phase	0	1
GO:0044784	metaphase/anaphase transition of cell cycle	0	1
GO:0000075	cell cycle checkpoint	0	2
GO:0005618	cell wall	5	7
GO:0042546	cell wall biogenesis	5	5
GO:0071554	cell wall organization or biogenesis	10	6
GO:0070726	cell wall assembly	0	1
GO:0030243	cellulose metabolic process	2	5
GO:0005886	plasma membrane	17	28

Total of 21 GO accessions related to microtubule were revealed, including processes of microtubule binding, motor activity, microtubule anchoring, microtubule polymerization and depolymerization, regulation of polymerization and depolymerization, and organization of microtubule cytoskeleton and organizing center. Total of 16 DEGs were descripted as microtubule-related genes, in which 7 DEGs were up-regulated, 9 DEGs were down-regulated. For almost every GO accession of microtubule, the number of up-regulated DEGs was less than that of down-regulated DEGs. Four GO accessions related to motor activity were scanned out, one up-regulated and three down-regulated DEGs. Total of 6 unigenes were spindle-related DEGs, playing roles in 7 GO accessions including spindle assembly, organization, attachment of spindle microtubules to kinetochore, etc. Three up-regulated and 5 down-regulated kinetochore-related DEGs were uncovered, and involved in 10 GO accessions. Two down-regulated cell cycle checkpoint DEGs were involved in one GO accession. Total of 14 Golgi-related GO accessions were revealed, 4 up-regulated and 10 down-regulated DEGs, which were described as Golgi membrane, Golgi transport, etc. The transport and organization of vesicle contained 19 up-regulated and 33 down-regulated DEGs, which were involved in 20 GO accessions. Eight up-regulated and 10 down-regulated segregation-related DEGs distributed in 17 GO accessions, which were involved in nuclear division, sister chromatid segregation, mitotic cytokinesis, etc. In addition, number of up-regulated DEGs in each of these 17 GO accessions was less than that of down-regulated DEGs, except 3 GO accessions of nuclear division.

For DEGs involved in the GO accessions mentioned, the fold changes of the 16 microtubule-related genes ranged from −5.2 (down-regulated) to 8.9 (up-regulated), most of them were up- or down- expressed at a level from 1 to 2. However, these 16 DEGs were expressed 3.4 fold low to 3.4 fold high between DacR5tr and DacR0. In the microtubule-related genes, 4 of 16 DEGs were found to regulate the motor activity according to GO accessions, which were related to movement in cell, and displayed 5.2-, 1.3-, 1.2-fold down- and 1.2- fold up-regulated expression. For the spindle-related DEGs, 3 genes were 1.1-, 1.3- and 1.9-fold down-regulated, while another 3 genes were 1.5-, 1.7- and 3.4-fold up-regulated. The up-regulated genes that showed higher fold change were involved in spindle organization and assembly. The organization of spindle microtubule and its attachment to kinetochore were in the 3 down-regulated spindle DEGs. Analysis of the kinetochore DEGs showed that fold changes of 5 down-regulated DEGs ranged from −5.1 to −1.1, while fold changes of other 3 DEGs were from 1.1 to 2.0 up-regulated expression. Two DEGs in cell cycle checkpoint (GO: 0000075) were down-regulated with about 2.7- and 1.5-fold change. A lot of membrane and vesicle assembly and transport DEGs were differentially expressed, including 34 down-regulated DEGs and 20 up-regulated DEGs with fold change from −6.3 to 6.3. According to the GO description, only one gene was involved in the process of sister chromatid segregation (GO: 0000819 and 0033045), which was 1.0- fold down-regulated in DacR5tr vs DacR5ck, 1.4-fold down-regulated in DacR5tr vs DacR0.

### Validation of the DEGs

To determine a reference gene for quantitative PCR (qPCR) analysis, three candidate genes, β-actin, ABC (ATP-binding cassette sub-family F member) and EF1 (elongation factor 1), were tested using qPCR and their expression stability was compared using Bestkeeper ver. 1.0^32^, NormFinder ver. 0.953^33^ and GeNorm ver. 3.5^34^. The β-actin gene, c83951_g1, was chosen as reference gene because it was the most stably expressed one among the tested genes.

To validate the expression patterns of DEGs from transcriptomes, total of 11 from 19 DEGs were finally characterized by qPCR using samples of *Dactylis* accession PI 441032 and PI 237607 under 5 h water (5hck) and colchicine (5htr) treatment (Table 4). According to gene function annotation, these genes were described as positive regulation of cell proliferation (c100325_g1), tubulin alpha (c101064_g1), attachment of spindle microtubules to kinetochore (c102023_g1), oxidation-reduction process (c96405_g1), regulation of transcription (c101317_g1), photosystem II assembly (g93529_g2), mRNA splicing (c49608_g1), membrane (c71496_g1), cell motility (c89024_g1), kinetochore protein (c101667_g3) and aquaporin NIP (c88478_g2). Compared with the 5hck of PI 441032, the expressions of c100325_g1, c101064_g1, c102023_g1, c96405_g1, c101317_g1, c93529_g2 and c49608_g1 were up-regulated, while c71496_g1, c89024_g1, c101667_g3 and c88478_g2 were down-regulated in 5hTR of PI 441032. The expression patterns of these DEGs showed the same trends to the data from transcriptomes. Furthermore, almost the same expression trends of the DEGs were detected in 5htr vs 5hck of PI 237607.

**Table 4:** Real-time PCR validation of transcriptome data.

Gene ID	Gene function annotation	Expression pattern		
		DacR5htr vs DacR5hck	5htr vs 5hck of PI 441032	5htr vs 5hck of PI 237607
c100325_g1	positive regulation of cell proliferation	+	+	+
c101064_g1	Tubulin alpha	+	+	+
c102023_g1	attachment of spindle microtubules to kinetochore	+	+	+
c96405_g1	oxidation-reduction process	+	+	+
c101317_g1	regulation of transcription	+	+	+
g93529_g2	photosystem II assembly	+	+	+
c49608_g1	mRNA splicing	+	+	+
c71496_g1	membrane	−	−	−
c89024_g1	cell motility	−	−	+
c101667_g3	kinetochore protein	−	−	−
c88478_g2	aquaporin NIP	−	−	+

Total of 11 DEGs were detected by qPCR to validate their expression patterns which were revealed by data of transcriptomes. The expression patterns of DEGs were presented by transcriptomes of DacR5htr vs DacR5hck, and were validated via qPCR in samples of 5htr vs 5hck of PI 441032 and PI 237607. Symbols were used to represent the expression patterns with “+” meaning up-regulation and “−” meaning down-regulation.

**Figure 2:**
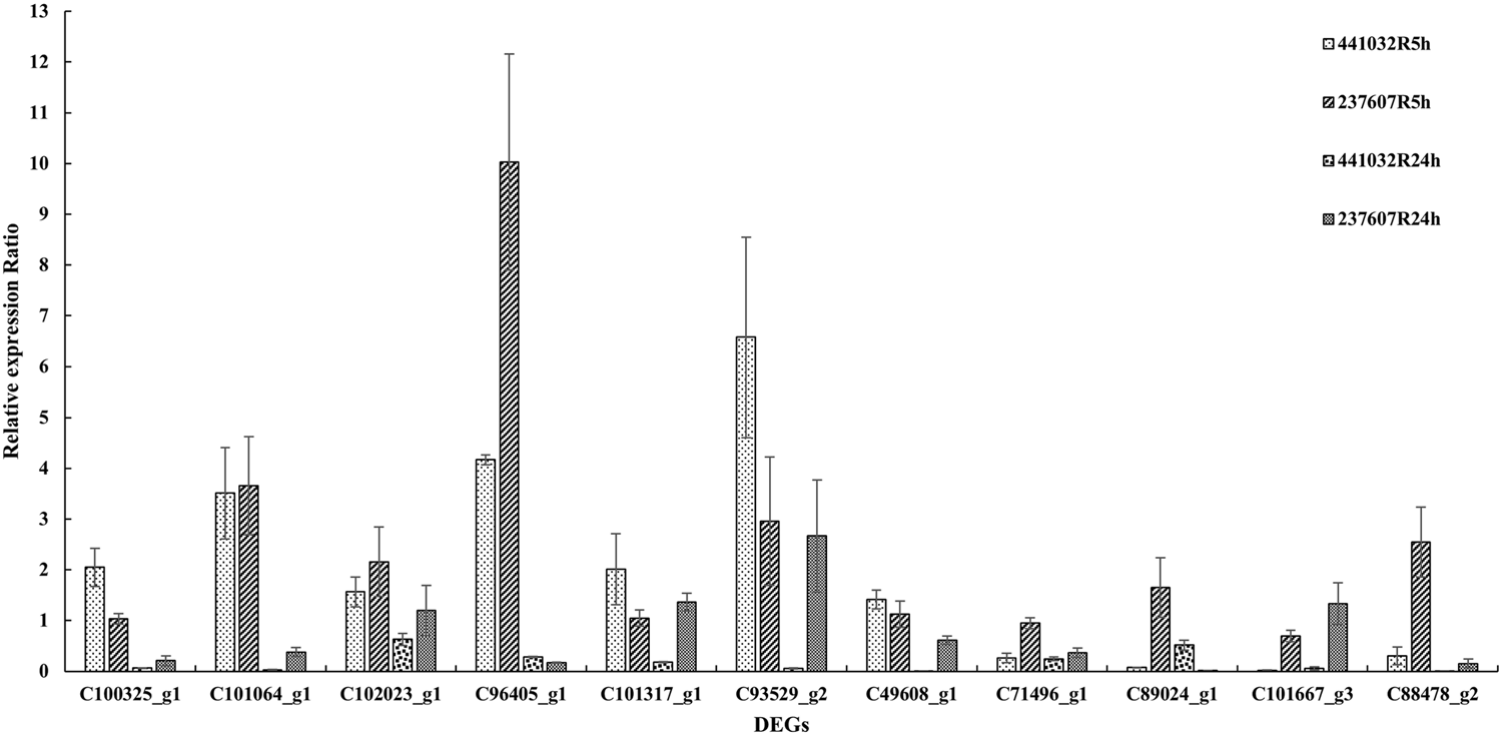
Relative expression of DEGs by qPCR. Mean relative expression ratio with standard deviation was calculated with the data from qPCR with β-actin as reference gene. 4 sets of comparisons were analyzed which were colchicine-treated sample versus water-treated sample of PI 441032 and PI 237607 under 5 h treatment and 24 h treatment.

In 5hTR of the accession PI 237607, the expression level of c100325_g1, c101064_g1, c102023_g1, c96405_g1, c101317_g1, c93529_g2 and c49608_g1, were higher, while c71496_g1 and c101667_g3 were lower than that in the 5hck. Nine of the 11 differentially expressed genes in 5htr vs 5hck of PI 237607 were detected with the same expression trend as those in PI 441032. However, the c89024_g1 and c88478_g2 genes showed different expression trends in these two accessions, which were down-regulated in PI 441032 but up-regulated in PI 237607 after treatment (Fig. 2). To reveal the gene expression changes under a long-time treatment, the 11 DEGs were also tested in samples of PI 441032 and PI 237607 under 24 h treatment of water (24 hck) and colchicine (24htr). After 24h treatment, all of the genes were down-regulated in the PI 441032, while most of the genes were down-regulated in the PI 237607 (Fig. 2).

## Discussion

Colchicine is one antimitotic agent, and used frequently for chromosome doubling. However, it is also a toxic chemical, possessing great threat to normal plant life. In treatment of *Platanus acerifolia* using colchicine, higher frequency of chromosome doubling was correlated with higher concentration of colchicine, but accompanied with a lower survival rate. Under colchicine treatment, survival rate of *Miscanthus* plants significantly depended on each genotype, colchicine concentration and exposure time^35^. High polyploidization rate and high lethality prompted us to select a suitable treatment method^16, 35–37^. In our study, 2.5 mM colchicine (0.1%, w/v) and 2% DMSO was used to characterize the changes of genes expression in response to colchicine treatment for 5 h and 24 h.

To double the chromosome of plants using colchicine, scientists often soaked plant roots in colchicine solution^16, 31^. Effects on treated plants were from water, colchicine and potential synergism of water and colchicine, which could cause transcriptome changes. In our study, we analyzed the transcriptome of *D. glomerata* subsp. *smithii* under 0 h treatment, 5 h water treatment and 5 h colchicine treatment. 3381 DEGs were affected by water. Compared with DacR0, 3582 DEGs were screened out from DacR5tr which was treated by colchicine solution, thus these DEGs were mainly affected by water, colchicine, and potential synergism of water and colchicine. In comparison of DacR5tr with DacR5ck, 2247 DEGs were identified to be mainly affected by both colchicine and potential synergism of water and colchicine. Comparing these 2247 DEGs with DacR0, 976 were significant differentially expressed in DacR5ck under water stress, and were expressed with high or low level in DacR5tr after colchicine was added (Fig. 1e). These DEGs were affected by water or colchicine. The remaining 1258 DEGs that were expressed significantly in DacR5tr but not in DacR5ck, were considered to be mainly affected by colchicine and potential synergism of water and colchicine.

Colchicine is a bioactive alkaloid, one of the antimitotic agents. Its effect on human health and diseases was frequently reported. With suitable dosage, colchicine has been used to inhibit cancer, induce apoptotic cell death, and as an anti-inflammatory for disrupting tubulin. Its effect on the immune system included inhibition of neutrophil chemotaxis, superoxide production, etc^24^. Its well-known effect on plant cells was only its inhibition of microtubules in mitosis^38, 39^. In our study, transcriptomes were analyzed to explore its effects on gene expression. According to GO analysis, DEGs were mainly enriched in 16 GO terms with data of DacR5tr vs DacR5ck. DEGs included in these 16 GO terms had lower or higher expression in DacR5tr than in DacR5ck, whose changes were mainly affected by colchicine. Specially, DEGs related to cation binding especially metal ion were induced to a high level of expression, while more DEGs related to the other GO terms were restrained at low level expression under colchicine stress, including the major terms of catalytic activity, membrane, transporter activity, etc. The genes in KEGG pathways of phenylpropanoid biosynthesis, phenylalanine metabolism, and plant hormone signal transduction were mainly down-regulated, while genes in metabolism processes of starch, sucrose, cysteine, methionine, galactose, and chemical carcinogenesis were up-regulated. Phenylpropanoids biosynthesize the amino acids of phenylalanine and tyrosine, and are found throughout the plant kingdom. Phenylpropanoids are not only indispensable for structural polymers, but also involved in responses to biotic or abiotic stresses^40^. Plant hormones play an important role in regulating plant growth and development by the transmission of molecular signals in cells^41, 42^. Decrease in expression of genes related to phenylpropanoid, phenylalanine, plant hormone and increase in expression of more chemical carcinogenesis genes caused by colchicine might partly explain why high mortality rate was accompanied with chromosome doubling when colchicine was used.

Mitosis involves replication of chromosomes and division of the mother cell into two daughter cells leading the individual to grow^22, 43^. In the mitotic process of plant cells, organization of microtubule, motor protein, spindle, chromosomal kinetochore and vesicle play important roles, while several processes of movement of cytoplasm, segregation of chromatids, check of metaphase, development of membrane and cell wall around the cell plate in middle of cells, decide the final cell division^44, 45^. In our research under colchicine treatment, more than 170 GO terms were found to be related to microtubules, motor proteins, kinetochores, cytokinesis, etc. According to previous studies, assembly of microtubules was inhibited by binding of colchicine with alpha- and beta-tubulin dimers, without reducing disassembly process^46^. Assembly and depolymerization of microtubules were less frequent than its disassembly, resulting in the reduction of microtubules. However, in our research, more genes in GO terms related to microtubules, microtubule binding, tubulin complexes and microtubule associated complexes were down-regulated, while GO terms with more up-regulated genes were mainly related to microtubule anchoring, cytoskeleton and organizing center. Total of 6 spindle-related genes were found, including 3 up-regulated and 3 down-regulated genes. Gene expression for assembly of spindle was increased, while genes for spindle microtubule and its attachment to kinetochore were restrained. Thus, colchicine influenced the microtubule and spindle by not only reducing density of available tubulin dimers but also regulating the related genes’ expression. The expression of genes related to segregation of sister chromatids was down-regulated, resulting in inhibition of sister chromatid segregation under colchicine treatment. The metaphase checkpoint guarantees the exact segregation of chromosomes and promotes cell cycle to anaphase^47^. However, the expression of checkpoint genes was down-regulated by colchicine, which possibly leads the cell to proceed to anaphase unsuccessfully. Furthermore, inhibitions caused by colchicine also occurred in movement of cell components and cytokinesis. Relatively, the cell plate and cell wall in the middle of the cell was not easy to be formed with less vesicles and less biogenesis of cellulose under the inhibition of colchicine. As a result, the cell division cannot be completed under colchicine.

In our study, water stress was actually a waterlogging stress. Under waterlogging, the plant encounters the condition of hypoxia (deficiency of O_2_) or anoxia (absence of O_2_). Growing in oxygen deficient conditions, plant cell activity was energized by root anaerobic respiration. As a result, ethanol was accumulated. Furthermore, it was reported that anaerobic polypeptides (APNs) including lactate dehydrogenase and alcohol dehydrogenase (ADH) were increased under low oxygen environment as ADH was the major APNs^48^. Total of 26 ADH DEGs from water stressed *Dactylis* were expressed, 8 down-regulated and 18 up-regulated. However, 16 of the 26 ADH DEGs were down-regulated under colchicine treatment in comparison with DacR5ck. The highest expression level of ADH was 10-fold up-regulated in DacR5ck vs DacR0, while it was only 3-fold up-regulated in DacR5tr vs DacR0, suggesting that colchicine might relieve the stress from waterlogging. Moreover, KEGG pathways showed that more apoptosis related genes were down-regulated in DacR5tr than those in DacR5ck. In this study, treatment was performed with 2.5 mM colchicine (0.1%, w/v) mixed with 2% dimethyl sulfoxide for 5 h. Compared to previous studies^49, 50^, concentration of colchicine used in our treatment was low, and treatment time was short, which may be moderate to orchardgrass young plants. In addition, our results suggested that colchicine restrained the microtubules and inhibited the gene expression of cytokinesis, which might slowdown the cell activity, delay the cell into anaerobic respiration, resulting in apoptosis at a late stage and relieving the stress from waterlogging.

In summary, the inhibition of colchicine on microtubules was suggested by gene expression. Numbers of genes related to spindle, chromosomal kinetochore, vesicle, cellulose and processes of cytoplasm movement, chromatid segregation, membrane and cell wall development were also affected by colchicine. Multiple organizations and processes worked together to prevent cell division. Our results suggested that colchicine might have a buffer effect on relieving waterlogging stress. For further research, we suggested that solid medium mixed with colchicine be used for chromosome doubling to eliminate the effect caused by water, such as agarose culture medium, in this case, how genes respond to colchicine treatment during chromosome doubling can be precisely figured out.

## Methods

### Plant materials and treatment

Seeds of diploid plant of *D. glomerata* subsp. *smithii* Link (2n = 2x = 14) were kindly provided by the GRIN (USDA-ARS Germplasm Resources Information Network). Two accessions, PI 237607 from Spain and PI 441032 from United Kingdom, were used. Seeds were germinated under room temperature, and transplantation of seedlings was followed after plant expanding with 3–4 leaves. Seedling plants grew in greenhouse with regular irrigation. Plants were identified via observation of morphology and cytology.

After one month, young plants were used for treatment. To keep the plants away from physical damage or at least minimize the physical damage during collection, whole young plants with soil around the roots were moved softly from the container. Soil was washed off softly with running water. Dozens of clean plants were separated randomly into 2 groups after their roots were cut off about 3 cm from the bottom. One of the groups grew in water, whereas another one was treated by 2.5 mM colchicine (0.1%, w/v) mixed with 2% dimethyl sulfoxide (DMSO). Roots of samples in each group were separated randomly into more than 3 sub-groups, each with a few dozen of plants. Roots tissues were harvested from each sub-group at the time of 0 h, 5 h and 24 h, and preserved immediately in liquid nitrogen for RNA isolation. After treatment, all of the samples were transplanted and grew in greenhouse for seeds setting, identification and generating of chromosome doubled plants as new germplasm resource for *Dactylis* breeding.

### RNA isolation and cDNA preparation

Total RNA from root tissues was isolated using TRI Reagent® (Sigma-Aldrich, USA) following manufacturer’s protocol. To eliminate the potential contamination from genomic DNA, RNAs were treated by RNase-free DNase set (QIAGEN, USA). RNA quality was evaluated using electrophoresis on 1.0% agarose gels, while quantity was measured using NanoDrop 2000 Spectrophotometer (Thermo Scientific, USA). cDNA was synthesized using QuantiTect Reverse Transcription Kit (QIAGEN, USA). Total RNA and cDNA of each accession under treatments were prepared and used for Solexa sequencing and real-time PCR, respectively. Three RNAs samples of accession PI 441032 were sequenced using Solexa sequencing, and were named as DacR0 (0 h, non-treated), DacR5ck (5 h, water treated) and DacR5tr (5 h, colchicine treated). The cDNAs of 5 h water-treated and 5 h colchicine-treated samples of PI 441032 were used to validate differentially expressed genes identified in transcriptomic analysis using qPCR. The 24 h water-treated and 24 h colchicine-treated samples of PI 441032 were used to observe trends in gene expression in a longer colchicine treatment time. The cDNAs of 4 samples of PI 237607 were used to examine if the gene expression trend in this accession was similar to those in the PI 441032 using qPCR.

### Sequencing and gene annotation

For each sample, at least 10 μg of total RNA with A260/A280 > 2.0, A260/A230 > 2.0 was sent to the Novogene (Beijing, China) for Solexa sequencing commercially. The mRNA was purified from total RNA using oligo (dT) magnetic beads after quality control. Synthesis of cDNA was processed via steps of fragmentation of mRNA, the first strand was synthesized using random hexamer primer and reverse transcriptase, and the second strand was synthesized using RNase H and DNA polymerase I. Final cDNA library was harvested after terminal repair, size selection, adapter ligation, PCR amplification and purification. Library quality was evaluated on Agilent Bioanalyzer 2100 system (Agilent Technologies, USA). The index-coded samples were clustered using TruSeq PE Cluster Kit v3-cBot-HS (Illumia) on cBot Cluster Generation System. Prepared cDNA library was fed into HiSeq platform and paired-end reads were generated.

Raw reads were obtained from the sequencing-received image data, and then clean reads were filtered by discarding adapter-containing, poly-N-containing and low-quality reads through in-house perl scripts. Q20, Q30, GC-content and sequence duplication were calculated for clean data. Because of the absence of a reference genome, the transcriptome was assembled from clean reads using Trinity^51^ with min_kmer_cov set to 2 and the other parameters at default.

Gene functional annotation was achieved after BLAST searching in seven databases. The seven databases and their corresponding e-value threshold were as follows: Nr (NCBI non-redundant protein sequences, e-value = 1e-5), Nt (NCBI nucleotide sequences, e-value = 1e-5), Pfam (Protein family, e-value = 0.01), KOG/COG (Clusters of Orthologous Groups of proteins, e-value = 1e-3), SwissProt (A manually annotated and reviewed protein sequence database, e-value = 1e-5), KEGG (Kyoto Encyclopedia of Genes and Genome, e-value = 1e-10), GO (Gene Ontology, e-value = 1e-6).

### Identification and enrichment analysis of DEGS

To quantify the gene expression levels for each sample, clean data was mapped back to the assembled transcriptome, read count for each gene was obtained from the mapping results. All these were performed using RSEM^52^. Subsequently, the read counts were adjusted by edgeR program package through one scaling normalized factor. DEGs were respectively screened out from comparisons of DacR5ck vs DacR0, and DacR5tr vs DacR5ck by DEGseq (2010) R package with parameter setting as q-value < 0.005 and|log2 (fold change)| > 1.

Differentially expressed genes were submitted to GO term for enrichment analysis using the GOseq R packages based on Wallenius non-central hyper-geometric distribution^53^. The DEGs were also applied to KEGG pathway enrichment analysis implemented by KOBAS^54^ to test the statistical enrichment of DEGs in KEGG pathways.

### Detection for gene expression with qPCR

Total of 19 unigenes were randomly chosen for validating the expression of DEGs presented in the transcriptomes and detecting the variation of their expressions in samples using qPCR. Primers for unigenes were designed by Primer3 version 0.4.0^55^ and Beacon designer version 8.14 (PREMIER Biosoft, Palo Alto, CA, USA), suitable primers were screened out for the final qPCR experiment by estimating its specificity, amplification efficiency, etc. The cDNA of each sample prepared was diluted 1:10 with nuclease-free water before qPCR analysis. Each reaction was performed in total of 15 μl mixture containing 7.5 μl of 2 × SYBR® Green ROX qPCR mastermix (QIAGEN, USA), 1 μl of cDNA template, 0.5 μM of each primer, with nuclease-free water supplied to final volume. The qPCR was carried out in 96-well blocks with ABI PRISM® 7000 Sequence Detection System (Applied Biosystems). qPCR amplification was performed under a program: 10 min at 95 °C, followed by 40 cycles of 95 °C for 15 s and 60 °C for 1 min. Dissociation curve was obtained by heating the amplicon from 60 to 95 °C. Technical and biological triplicates were applied for qPCR reaction. Mean relative expression ratio was calculated using the 2^−ΔC(t)^ method^56^ with one stably expressed gene as reference gene.
